# Healthy human CSF promotes glial differentiation of hESC-derived neural cells while retaining spontaneous activity in existing neuronal networks

**DOI:** 10.1242/bio.20134648

**Published:** 2013-05-13

**Authors:** Heikki Kiiski, Riikka Äänismaa, Jyrki Tenhunen, Sanna Hagman, Laura Ylä-Outinen, Antti Aho, Arvi Yli-Hankala, Stepani Bendel, Heli Skottman, Susanna Narkilahti

**Affiliations:** 1 Critical Care Medicine Research Group, Department of Intensive Care Unit, Tampere University Hospital, FI-33521 Tampere, Finland; 2 NeuroGroup, Institute of Biomedical Technology/BioMediTech, University of Tampere, FI-33520 Tampere, Finland; 3 Department of Surgical Sciences, Anaesthesiology and Intensive Care, Uppsala University, SE-751 85 Uppsala, Sweden; 4 Neuroimmunology Unit, Medical School, University of Tampere and Tampere University Hospital, FI-33014 Tampere, Finland; 5 Coxa – Hospital for Joint Replacement, FI-33520 Tampere, Finland; 6 Department of Anesthesia, Tampere University Hospital, FI-33521 Tampere, Finland; 7 Department of Intensive Care Medicine, Kuopio University Hospital, FI-70029 Kuopio, Finland; 8 Opthalmology Group, Institute of Biomedical Technology/BioMediTech, University of Tampere, FI-33520 Tampere, Finland; 9 The Science Centre of Pirkanmaa Hospital District, FI-33521 Tampere, Finland

**Keywords:** Astrocyte, Microelectrode array, Network activity, Neuron, Oligodendrocyte, Stem cell

## Abstract

The possibilities of human pluripotent stem cell-derived neural cells from the basic research tool to a treatment option in regenerative medicine have been well recognized. These cells also offer an interesting tool for *in vitro* models of neuronal networks to be used for drug screening and neurotoxicological studies and for patient/disease specific *in vitro* models. Here, as aiming to develop a reductionistic *in vitro* human neuronal network model, we tested whether human embryonic stem cell (hESC)-derived neural cells could be cultured in human cerebrospinal fluid (CSF) in order to better mimic the *in vivo* conditions. Our results showed that CSF altered the differentiation of hESC-derived neural cells towards glial cells at the expense of neuronal differentiation. The proliferation rate was reduced in CSF cultures. However, even though the use of CSF as the culture medium altered the glial vs. neuronal differentiation rate, the pre-existing spontaneous activity of the neuronal networks persisted throughout the study. These results suggest that it is possible to develop fully human cell and culture-based environments that can further be modified for various *in vitro* modeling purposes.

## Introduction

Currently, human neural stem cells are considered to serve as a promising supply not only for tissue/cell transplantation but also for *in vitro* modelling of the nervous system, neurodevelopment studies, neurotoxicological screening of various substances, and drug screening and development (Gaspard and Vanderhaeghen, 2011; Ylä-Outinen et al., 2010). Human derived neural cells can be isolated from various sources including aborted fetuses, adult human brain, and post-mortem brain tissue (Palmer et al., 2001; Piao et al., 2006; Roy et al., 2000). Even though these sources provide potentially adequate material for such investigations, one could claim that neural cells obtained either from human embryonic stem cells (hESC) or from induced pluripotent stem cells (iPCS) could more readily serve this purpose (Carpenter et al., 2001; Karumbayaram et al., 2009; Takahashi et al., 2007; Thomson et al., 1998; Yu et al., 2007). This suggestion is based on the notion that the human pluripotent stem cells are the most proliferative cell type thus providing indefinite cell source. They can be efficiently differentiated into neural precursor cells that can be further differentiated into neurons (including specific subtypes), astrocytes, and oligodendrocytes (Itsykson et al., 2005; Lappalainen et al., 2010; Sundberg et al., 2010). These, mainly hESC-derived, neural cells have already been tested in transplantation experiments in animal models (Daadi et al., 2008; Hicks et al., 2009; Keirstead et al., 2005; Sundberg et al., 2011), in neurotoxicity testing (Ylä-Outinen et al., 2010; Zeng et al., 2006), and in development of efficient differentiation protocols (Lappalainen et al., 2010).

Obviously, the cell culture conditions have a huge effect on the survival, proliferation, differentiation, and to the functionality of human neural cells. The commercially available culture media typically contain substantial amounts of various factors that enhance neural differentiation or maintenance, in addition to various growth factors and enhanced buffering agents that are supplemented to the culturing media. Thus, the *in vitro* cultures significantly differ from the “natural” environment of the neural stem cells *in vivo* that includes extracellular matrix, intercellular fluids, and cerebrospinal fluid (CSF). When considering the use of *in vitro* cultured neural cells for e.g. *in vitro* modelling or neurotoxicity screening, the utilization of highly enriched culture medium may interfere the results considerably. Previously, it was shown that if adult human neural cells obtained during epilepsy surgery are cultured in human CSF (collected from normal pressure hydrocephalus patients), the neurospheres survive but proliferate less compared to cells cultured in the control media containing growth factors (Buddensiek et al., 2010). In addition, human CSF seems to promote astroglial differentiation instead of neurons (Buddensiek et al., 2010). Meanwhile, several reports describe the effects of human CSF on rodent or chicken-derived neural cultures. These studies report that CSF derived from: 1) ALS patients increase the viability of cells (Nagaraja et al., 1994), 2) epilepsy patients enhance the neurite outgrowth (Akoev et al., 1996), 3) MS patients induce cell death and inhibit proliferation (Cid et al., 2003; Westall and Seil, 2006), and 4) from traumatic injury patients inhibit neuronal network function (Otto et al., 2009) of animal derived neural cultures. However, currently there are no reports describing the effects of CSF derived from neurologically healthy individuals on the neural cultures.

In this study we developed a reductionistic model where hESC-derived spontaneously functional neuronal networks were cultured in artificial or human CSF from neurologically healthy individuals or in control media. The effects of CSF on the neural cells were monitored using microscopy, time-lapse imaging, proliferation analysis, immunostaining, and a micro electrode array (MEA) setup for 4 weeks.

## Results

### Human CSF supported neural cell growth whereas artificial CSF was detrimental to the cells

**Fig. 1.**
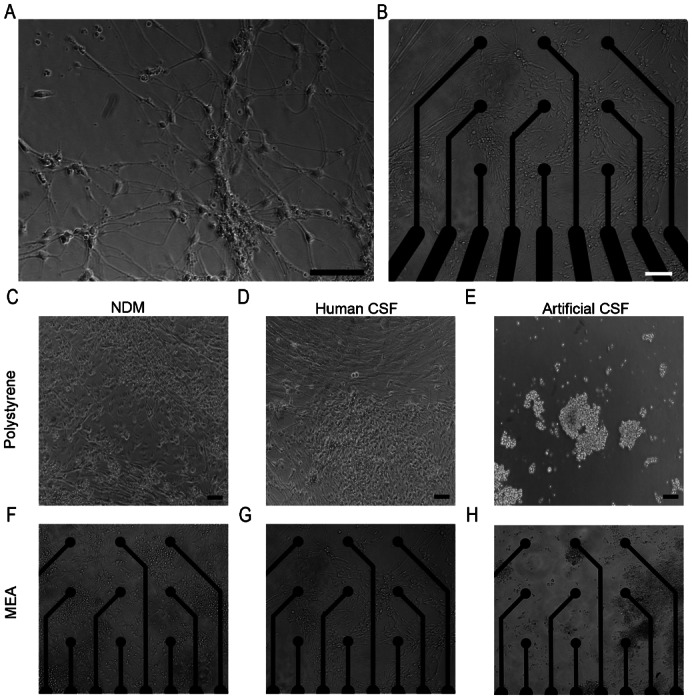
Neuronal cells grew well on (**A**) laminin-coated polystyrene and (**B**) PEI and laminin-coated MEA plates in NDM before the artificial or human CSF exposure. Two weeks after the artificial or human CSF exposure, neuronal cells grew in NDM (**C**,**F**) and human CSF (**D**,**G**) but detached in artificial CSF cultures (**E**,**H**). Scale bars: 100 µm.

After 8–15 weeks of differentiation the cell spheres were dissociated and replated in NDM on human laminin coated wells or MEA dishes. Most of the cells resembled morphologically bipolar neuronal cells and formed neuronal networks when investigated two days after replating (Fig. 1A,B) before artificial or human CSF exposure was initiated. After two weeks of exposure, cells grown in artificial CSF died and detached (Fig. 1E,H). Thus, cells grown in artificial CSF were not included in the following analysis. In contrast, the cells grown in human CSF from neurologically healthy individuals (Fig. 1D,G) or NDM (Fig. 1C,F) remained viable, although the cells grown in CSF reacted to the CSF change at 1 week time point after which recovery and increased gliogenesis was observed. The result was the same regardless of whether the cells were plated as single cells or aggregates (data not shown).

### Neuronal cells proliferated in both control medium and human CSF

**Fig. 2.**
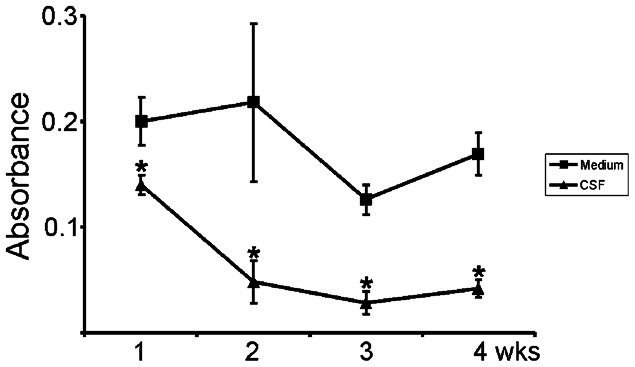
Proliferation of neural cells in NDM or CSF during follow-up of 4 weeks. Cells cultured in NDM proliferated efficiently throughout the follow-up period whereas the proliferation of cells in human CSF was significantly decreased at all time points studied (*P<0.001) compared to control cultures.

During the four weeks' follow up, the cells grown in CSF and NDM were both proliferative. The BrdU analysis showed, however, more pronounced proliferation in the neural networks cultured in NDM than in CSF at all time-points (*P*>0.001 in all studied time points) (Fig. 2).

### Human CSF promotes glial differentiation over neuronal differentiation

**Fig. 3.**
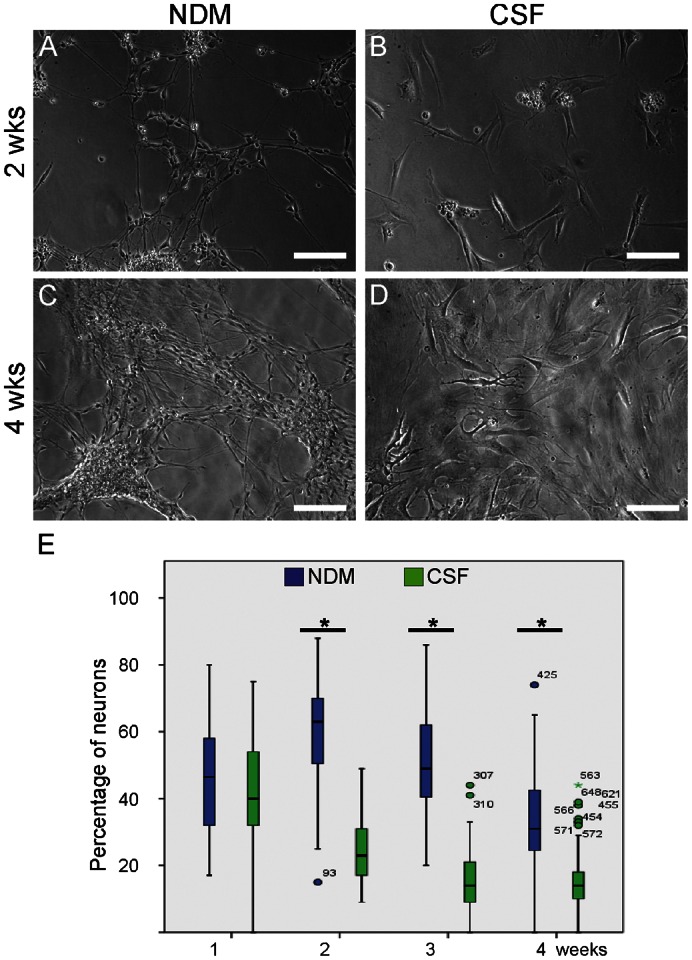
Morphology of the cells cultured in NDM remained neuronal throughout the follow-up period (**A**,**C**) but turned into glial-like cells when cultured in human CSF (**B**,**D**). (**E**) The number of neuronal cells cultured in medium or in human CSF during 4 weeks follow-up. The cells were imaged for 48 hours in each time point after which at least 10 of 500×670 µm size images/condition were automatically imaged with design protocol. In total, at least 200 cells/case were automatically analyzed and taken into statistical analysis. The box represents median and upper and lower quartiles, and the bars indicate minimum and maximum values. At time points 2, 3 and 4 weeks there were significantly less neuronal cells detected in CSF cultures when compared to NDM cultures (*P<0.05). Scale bars: 100 µm.

Time-lapse imaging data clearly showed morphological differences between cells cultured in human CSF or NDM. While the cells grown in NDM appeared to have neuronal-like morphology (Fig. 3A,C), the cells grown in human CSF expressed more pronounced glial cell-like morphology during the follow-up of four weeks (Fig. 3B,D). The time-lapse monitoring and analysis supported the visual inspection. According to the analysis, after one week approximately half of the cells grown in CSF or NDM were characterized as neurons (45.8% neuronal and 54.2% glial in NDM vs. 42.1% neuronal and 57.9% glial in human CSF) (Fig. 3E). During the second week, significantly more neurons were growing on NMD compared to CSF (59% neuronal in NDM vs. 24.0% neuronal in CSF, *P*<0.005) and thus more glial cells were growing in CSF than in NDM (41% glial in NDM vs. 76% glial in CSF). During the third week in CSF culturing the percentage of glial cells increased but in NDM the relation of neuronal and glial cells stayed approximately the same (51.1% neuronal and 48.9% glial in NDM vs. 18.3% neuronal and 81.7% glial in CSF, *P*<0.005). The status was the same also during the fourth week (45.3% neuronal and 54.7% glial in NDM vs. 15.5% neuronal and 84.5% glial in CSF, *P*<0.005).

**Fig. 4.**
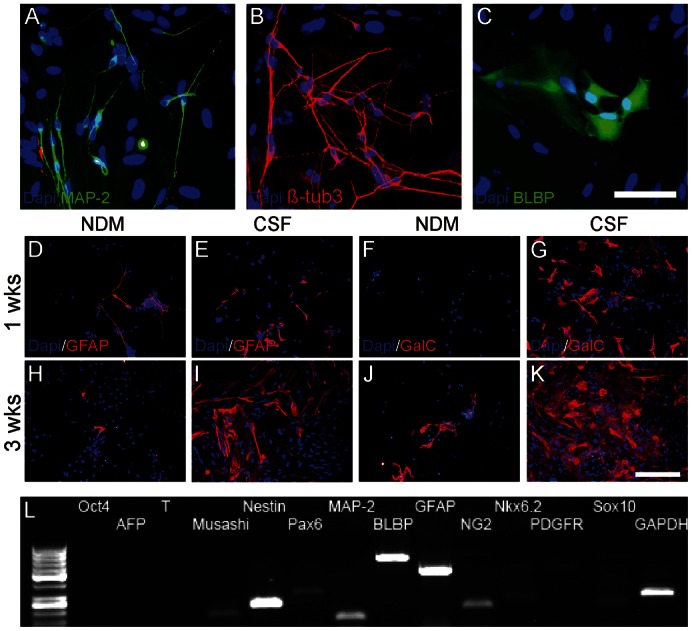
Representative pictures of MAP-2- and β-tubulin_3_-positive neuronal cells (**A**,**B**) and BLBP-positive radial glial cells (**C**) in CSF cultures. Cells cultured in NDM expressed glial proteins for astrocytes (GFAP) but not for oligodendrocytes (GalC) at first week's time point (**D**,**F**) and only a few glial cells could be detected at third week (**H**,**J**). In comparison, both GFAP- and GalC-positive glial cells were detected in CSF cultures already at first week (**E**,**G**) and the amount of glial cells increased towards the third week (**I**,**K**). Cells in CSF cultures expressed oligodendrocytical genes *NG2*, *Nkx6.2*, *PDGFR*, and *Sox10* in addition to other neural markers after 4 weeks of differentiation (**L**). Scale bars: 50 µm in A–C; 100 µm in D–K.

The immunocytochemical analysis verified the results gained from time-lapse imaging. MAP-2- and β-tubulin_3_-positive neuronal cells and BLBP-positive radial glial cells could be detected in CSF cultures throughout the follow-up time of 4 weeks (Fig. 4A–C). However, already at the first week, GFAP- and GalC-positive cells were detected in CSF cultures whereas NDM cultures did not contain GalC-positive cells at this time point (Fig. 4E,G vs. Fig. 4D,F). At later time-points, the number of glial cells (GFAP-positive and GalC-positive cells) increased in CSF cultured cell populations compared to NDM cultured cells (Fig. 4I,K vs. Fig. 4H,J) which was confirmed with manual cell counting (data not shown). The presence of both neuronal and glial cells in CSF cultures was confirmed with gene expression analysis after four weeks of differentiation and at this time point the cells expressed genes related to neural precursor, neuronal, astrocytical and, oligodendrocytical phenotype (Fig. 4L).

### Both human CSF and NDM supported the formation of spontaneously functional neuronal networks

**Fig. 5.**
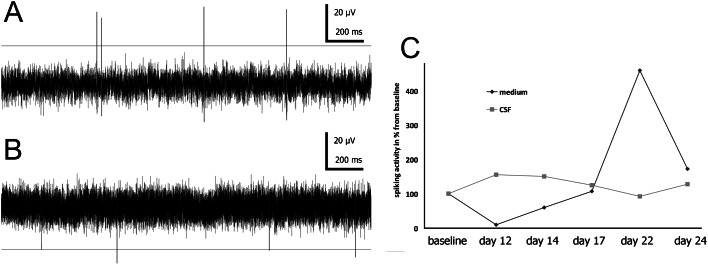
Spontaneous activity of neuronal networks in CSF (**A**) and NDM (**B**) after 17 days of culturing on MEA. Spiking activity from each measurement point is collected to graph (**C**). Activity is normalized against baseline (3 days on MEA) and showed as percentage change from baseline activity at each time point.

The cells were let to attach to MEA plates for three days prior to switch to artificial CSF, human CSF, or NDM, respectively. Baseline activity of the neuronal networks was measured in NDM prior to artificial or human CSF exposures which verified that MEA cultured networks showed initiation of functional activity detected as single spikes. As the cells grown in the artificial CSF did not survive, this group was excluded from the monitoring and the analyses. For the cells grown in human CSF or NDM, the MEA measurements were performed twice a week for three additional weeks. Neuronal networks on MEA in CSF or NDM are shown in Fig. 1F,G. In both groups, cells formed neuronal networks which showed spontaneous activity of spikes in several channels (Fig. 5A,B). In NDM, the activity of the networks increased temporarily during the follow-up period but in CSF the activity increment was not observed. Fig. 5C shows the electrical activity of NDM-MEAs and CSF-MEAs.

### Luminex analysis revealed presence of four growth factors FGF-2, B-NGF, PDGF-BB and VEGF-A in the human CSF

We showed that cells differentiated towards the glial cell lineages when cultured in human CSF. To understand whether the growth factors, that are present in the CSF, could partially explain this event, we analyzed the levels of six growth factors in the pooled human CSF by Luminex methodology. Analysis showed that four of the six growth factors were present in the CSF, although the levels were substantially low (FGF-2: 20.5 pg/ml, B-NGF: 12.6 pg/ml, PDGF-BB: 23.8 pg/ml and VEGF-A: 8.6 pg/ml). Two of the growth factors BDNF and EGF were not present in the CSF or were under detection limit. As a comparison, these growth factors were also analyzed from the control cells grown in NDM and none of these factors were detected.

## Discussion

The aim here was to study whether hESC-derived neuronal cells and networks could be cultured in native CSF for further, more valid, disease (e.g. MS, subarachnoidal haemorrhages, traumatic brain injuries) modelling *in vitro*. If neuronal pathologic conditions are to be induced in *in vitro* cell culture models, for validity the platform should be close to the environment *in vivo*.

Herein, we have shown that human CSF supports survival of hESC-derived neural cells in prolonged cultures. To our knowledge, this is the first report describing fully “humanized” *in vitro* neural culture using hESC-derived cells. Our study shows that the hESC-derived neural cells cultured in healthy human CSF differentiated substantially more towards glial cell lineages when compared to typical control neural differentiation medium. Previous experiment using human adult neural stem cells (Buddensiek et al., 2010) also reported increased gliogenesis. Hence, this experiment cumulates evidence about the gliogenic properties of cerebrospinal fluid to human-derived neural stem cells. Although the CSF grown cultures contained more glial cells, the underlying neuronal network remained spontaneously active. Thus, this study suggests that more *in vivo*-like human-derived neural culture models can be developed for various *in vitro* modeling applications. Further studies are needed for standardization of such models.

In the present investigation, we first tested the viability of hESC-derived neural cultures in artificial or human CSF compared to control neural differentiation medium. The artificial CSF did not support the neural cultures for prolonged times and thus these cultures were lost in 2 week follow-up time. The composition of artificial CSF was similar to previously reported ones that are used to maintain the *in vitro* and organotypic cultures (Verkuyl and Matus, 2006). In a reported study, the hippocampal slices were cultured in artificial CSF only for hours (Verkuyl and Matus, 2006) instead of weeks as in our experiment in which it did not seem to support neural cell survival. Here, the human CSF used in our study was a pooled sample collection from neurologically healthy individuals undergoing elective orthopedic surgery in spinal anesthesia. These individuals did not have any history of neurological disease, CNS malignancy, neuroinflammatory diseases, or immunosuppressive medication. Thus, they were considered as healthy volunteers. The CSF pools were analyzed for glucose, lactate, protein concentrations, and for possible microbial contaminations which also stated that obtained CSF was “normal” when compared to standard values. The glucose concentration varied between 3.1–3.8 mM from pool to pool and was thus much lower when compared to high glucose content of the NDM (20 mM). We did, however, decide not to adjust the glucose concentration as we wanted to test as natural CSF as possible. During the four week follow up we could see that human CSF supported the cell cultures surprisingly well that is in line with previous study reporting similar results after shorter follow-up with adult human neural stem cells (Buddensiek et al., 2010). Of note is that Buddensiek and co-workers used the CSF from the patients with normal pressure hydrocephalus. In our study the cell survival was also associated with continuous but decreasing proliferation rate in the CSF cultures compared to NDM cultured cells. In addition, the concentrations of neural-related growth factors were analyzed with Luminex from the pooled CSF lots to give a general overview of the composition of CSF in healthy population. It is known that human CSF includes various compounds including growth factors such as FGF and NGF (Mashayekhi et al., 2010; Mashayekhi and Salehi, 2005) and we also verified low levels of these factors in the CSF. The concentrations of found growth factors FGF, NGF, PDGF-BB, and VEGF-A were lower than normally used in cell culturing applications but it should be marked that these growth factors were not present in control NDM, thus they could have an effect on profound glial differentiation detected in CSF cultures. Recently, it was shown that IGF-2 in CSF from glioblastoma patients has a stimulative effect on neural stem cell proliferation (Lehtinen et al., 2011), and it would be interesting to analyze the IGF-2 levels from the healthy patients' CSF in the future to see if IGF-2 was involved in the changes detected between CSF and NDM cultures.

The analyzed growth factors using Luminex show that FGF which is known to have an effect on glial differentiation (Krencik and Zhang, 2011) was found in the CSF. Similarly, PDGF-BB which might have similar functions as PDGF-AA (Rydziel et al., 1994) was detected in native CSF. PDGF-AA has been utilized as an important factor in oligodendrocytical differentiation of hESCs (Sundberg et al., 2009) thus PDGF-BB might have promoted the glial differentiation detected here in CSF cultures. Also, VEGF-A has already been connected to glial differentiation of neural progenitor cells (Kim et al., 2009; Mani et al., 2005). CSF offers very limited supply of nutrients to the cells and thus by means of nutrition CSF closely resembles artificial salt solution supplemented with low concentration of glucose. Hence, it is interesting that cells in artificial CSF did not survive and cells in CSF survived as functionally active neural network for an extended period of culturing. It is possible that one or many the of growth factors we detected in CSF have an essential role in cell survival, but one cannot bypass that CSF has, in addition, been reported to include many other factors (Zappaterra and Lehtinen, 2012) which might induce gliogenesis as detected in this study.

Here, the human CSF was added to neural cultures exhibiting already immature spontaneous activity indicating that very premature neuronal networks had already being formed. The spontaneous activity of the cultures was monitored for four weeks. During this time the control cultures showed pronounced network activity development as described earlier (Heikkilä et al., 2009) whereas CSF cultures expressed stable but continuous lower activity levels. Most likely this is due to the increased amount of glial cells in CSF cultures. Even though glial cells seem to be necessary in synaptic formation (Blackburn et al., 2009; Ullian et al., 2004), the timing when glial cells integrate with neuronal networks maybe crucial for proper network activity development also *in vitro*.

In this study the human CSF was added to neural cultures when neuronal networks were still developing. It is reasonable to suggest that the lower glucose concentration in CSF compared to NDM is one factor driving the glial differentiation of these cultures but this aspect should be studied in more detail in the future. As the neuronal cells were able to continue active signaling in CSF it appears that CSF can support the growth of neuronal cells. To our knowledge this is the first time it is shown that prolonged culturing, while maintaining electrophysiological functionality of the cells, is possible in fully human based *in vitro* model using healthy human CSF and hESCs.

Sufficient amounts of CSF is relatively hard to obtain for cell differentiation but the observed fact that extended cell culturing of neural stem cells is possible makes CSF an interesting option for fully human based disease-specific *in vitro* modeling. Next logical step is to study the effect of CSF in matured *in vitro* neuronal networks expressing synchronous bursting activity and see whether the differentiation or spontaneous activity would be influenced. CSF is a complex fluid with a wide range of physiological functions (Zappaterra and Lehtinen, 2012) and thus would also be worth testing whether CSF from CNS disease-affected individuals could be used in *in vitro* modeling applications.

## Conclusions

The use of components with human origin; human CSF and human-derived neural networks could offer reductionistic *in vitro* models for various purposes. Especially, the hESCs and iPSCs could be used for the production of disease models for various diseases of the CNS (e.g. MS, subarachnoidal haemorrhages, traumatic brain injuries) on a dish. Effects of the patient (disease)-specific CSF could be used for studying the disease onset, progression, or prevention *in vitro*. Herein we report for the first time the effects of CSF from healthy individuals on human stem cell-derived neural/neuronal cells in long term culture. This will serve as a starting point for disease specific testing. These kinds of *in vitro* studies are still currently in an early phase but using more sophisticated methods, such as micro electrode arrays, these *in vitro* models will have a strong role in future neuroscience.

## Materials and Methods

### Human embryonic stem cells

The hESC line used in this study was Regea08/023, derived, maintained, and characterized in Institute of Biomedical Technology (IBT) (Skottman, 2010) (European Human Embryonic Stem Cell Registry, http://www.hescreg.eu). IBT has the approval of the Institutional Review Board of Tampere University Hospital in Pirkanmaa Hospital District (Skottman R05116) to derivate, culture, and differentiate hESCs and license from National Supervisory Authority for Welfare and Health to do research on human embryos.

In brief, hESCs were maintained in an undifferentiated stage by culturing them in medium consisting of Knockout Dulbecco's Modified Eagle Medium supplemented with 20% Knockout Serum-Replacement, 2 mM GlutaMax (all from Gibco Invitrogen, Carlsbad, CA), 1% non-essential amino acids (Cambrex Bio Science, East Rutherford, NJ), 50 U/ml penicillin/streptomycin (Lonza Group Ltd, Switzerland), 0.1 mM 2-mercaptoethanol (Gibco Invitrogen), and 8 ng/ml basic fibroblast growth factor (bFGF; R&D Systems, Minneapolis, MN) on top of a human feeder cell layer (CRL-2429, ATCC, Manassas, CA). The medium was changed five times/week. The undifferentiated stage of hESCs was daily assessed according to their morphology, and periodically by immunocytochemical staining with antibodies against Nanog, Oct-4, SSEA-4, and Tra-1-60, and by embryoid body forming assay demonstrating their *in vitro* pluripotency. The karyotype of the cells remained normal throughout the study and the cultures were mycoplasma free.

### Neural differentiation

Neural differentiation of hESCs was performed as previously described (Lappalainen et al., 2010; Sundberg et al., 2009). Briefly, undifferentiated hESCs were mechanically dissected into aggregates of approximately 3000 cells and directly transferred into 6-well ultra-low attachment plates (Nunc, Thermo Fisher Scientific, Rochester, NY) in neural differentiation medium (NDM) consisting of 1:1 DMEM/F12 and Neurobasal media supplemented with 2 mM GlutaMax, 1×B27, 1×N2 (all from Gibco Invitrogen), 25 U/ml penicillin/streptomycin (Lonza Group Ltd), and 20 ng/ml bFGF (R&D Systems). The aggregates formed constant spheres in a week. These aggregates were mechanically cut in 2–6 spheres each week to keep the diameter less than 500 µm and thus most cells in contact with the medium. The aggregates were cultured for 8 to 15 weeks and half of the medium was changed three times/week. The cultures were tested and found mycoplasma free.

### Medium replacement with artificial CSF or CSF from neurologically healthy individuals

Artificial CSF was prepared as previously described (Verkuyl and Matus, 2006). Briefly, the stock solution (20×) consisting of NaCl (124 mM), KCl (2.5 mM), MgSO_4_*7H_2_O (1.5 mM), NaH_2_PO_4_ (1.25 mM), CaCl_2_*2H_2_O (2.5 mM) was prepared beforehand but glucose (10 mM) and NaHCO_3_ (25 mM) were always added to the final solution freshly prior to use. Human CSF was collected after obtaining an informed consent from healthy individuals (age range 25–85 years, 44% males and 56% females) undergoing spinal anesthesia for elective orthopedic endoprosthetic surgery [Ethical approval (Tenhunen R09006) by the Institutional Review Board of Tampere University Hospital in Pirkanmaa Hospital District]. Patients were excluded if they had any indication or history of a disease of the central nervous system (CNS), infection, or immunosuppressive medication. A written informed consent was obtained at the preoperative visit one to seven days before operation. Spinal anesthesia was performed with a 27-gauge spinal needle at the level of lumbar vertebrae 3–4 (L3–L4) or L4–L5 intervertebral space. Once a free flow of CSF was detected, 1–2 drops of CSF were wasted before collecting the CSF. The CSF was aspirated with a sterile 5-ml syringe, and the tip of the syringe was immediately closed with a sterile cap. The aspirated amount of CSF was 3 to 4 ml, *i.e.* the same amount of spinal anesthetics that was planned for spinal anesthesia. After aspiration of the CSF, the spinal anesthetic was injected in the subarachnoid space, and the needle was removed. Collected CSF was excluded in case of blood contamination of the sample. The syringe containing the CSF was thereafter given to research personnel, and the CSF processing was started. Each CSF batch was centrifuged (500 *g*, 10 min), the supernatant was transferred to clean Eppendorf tubes, and stored in −70°C. For cell culture purposes the CSF batches were pooled upon use each week (CSF from 4 to 8 patients pooled together). A pooled lot was always analyzed for glucose, lactate, and protein concentration in the Pirkanmaan Hospital District, Fimlab Laboratories, Tampere (Table 1) before adding it to the cells. In addition, samples were analyzed for microbial contaminations which were not found.

**Table 1.**
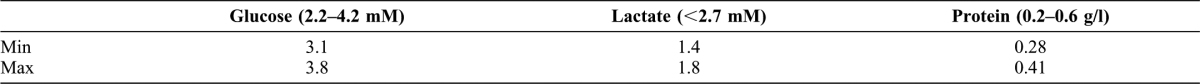
Results of the pooled CSF samples for glucose, lactate, and protein.

### Cell preparation

After differentiating the cells for 8–15 weeks the spheres (consisting mostly of neuronal cells and glial cells to some extent (Lappalainen et al., 2010)) were collected, either dissociated into singe cell suspension with TrypLE Select (Invitrogen) or manually dissected into small aggregates, and replated on human laminin (10 µg/ml; Sigma–Aldrich, St Louis, MO) coated 48-well plates in NDM (described above) without bFGF. The cells were allowed to attach and stabilize for three days after which the medium of 1/3 of the wells (at least 10 wells in each case) was replaced with artificial CSF, human CSF, or NMD (control), respectively and cultured for four weeks. Half of the volume of the artificial CSF, human CSF, or NDM was changed three times/week. Time-lapse imaging, proliferation measurement, and immunocytochemical analysis were performed each week. Cells were also plated on microelectrode array dishes (described below) and the networks' electrophysiological functionality was measured for up to four weeks. In addition, RT-PCR analysis was performed at the first and fourth week of the culturing.

### Measurement of cell proliferation

To analyze the cell proliferation we used an ELISA-based bromodeoxyuridine (BrdU) proliferation assay (Roche, Germany) as described earlier (Lappalainen et al., 2010). Briefly, the cells were incubated in BrdU labeling solution for 20 hours (4 wells/each condition) after which the cells were collected and replated on a 96-well plate, 5,000 cells in each well. Consequently, the cells were dried, fixed, and labeled with an anti-BrdU secondary antibody conjugated to peroxidase for 90 min. Subsequently, substrate solution was added for five minutes, colorimetric reaction was stopped with H_2_SO_4_, and the result was quantified with Victor^2^ 1420 Multilabel Counter (Perkin-Elmer Wallac) at the wavelength of 450 nm.

### Time-lapse imaging

Time-lapse imaging was performed with a Cell-IQ platform (Chip-Man Technologies Ltd, Finland). This platform enables controlled imaging of cells, e.g. human embryonic stem cells (Narkilahti et al., 2007) and hESC-derived neuronal cells (Lappalainen et al., 2010) with an indefinite amount of time in a thermal chamber with predetermined incubation gases (5% CO_2_) (Tarvainen et al., 2002). The well plate was transferred to Cell-IQ after changing artificial CSF, human CSF and NDM to the wells. The plate was monitored for 48 hours in every analyzed time point (0, 1, 2, 3, 4 weeks). The imaging positions were programmed in the Imagen software (Chip-Man Technologies), and every position was imaged in ∼30 min cycles. The imaging positions were programmed as 3×3 grids (2680 µm×2000 µm) in each well. The gained images were saved as jpeg-files and later analyzed using the Cell-IQ Analysis software (Chip-Man Technologies) to calculate the number of neuronal and glial cells.

### RT-PCR

A subpopulation of cells (2 wells in total for each condition) was collected in RA-buffer for RT-PCR analysis during the first and the fourth week of cell culturing in CSF or NDM. The RNA isolation was performed with the NucleoSpin XS RNA kit according to the manufacturer's instructions (Macherey-Nagel, Germany). The concentration and the purity of gained RNA was measured with NanoDrop after which 50 ng of RNA was taken to cDNA synthesis. CDNA was produced with a High Capacity cDNA reverse transcription kit as described in the manufacturer's instructions (Applied Biosystems, Carlsbad, CA). Next, PCR analysis was performed with the following genes: *Oct-4* for undifferentiated hESCs; *α-fetoprotein* for the endodermal lineage; *Brachyury/T* for the mesodermal lineage; *Musashi*, *Nestin*, and *Pax-6* for neural precursor cells; *microtubule associated protein* (*MAP-2*) for the neuronal cells; *brain lipid binding protein* (*BLBP*) for the radial glial cells; *glial fibrillary acidic protein* (*GFAP*) for the astrocytes; and *NG2*, *Nkx6.2*, *PDGFR*, and *Sox10* for the oligodendrocytes (for primer sequences see supplementary material Table S1). All primers were purchased from biomers.net GmbH (Germany). The PCR reaction consisted of 0.2 mM both forward and reverse primers, 1×PCR buffer (−MgCl, +KCl), 1.5 mM MgCl_2_, 0.1 mM dNTP mix, and Taq DNA polymerase (Qiagen). The cDNA was amplified using 35 PCR cycles containing an initialization step of 3 min at 95°C, DNA denaturation at 95°C for 30 s, annealing at 55°C for 30 s, and elongation at 72°C for 1 min. At the end, a 5-min elongation step finalized the reaction. The PCR samples were separated electrophoretically on a 1.5% agarose gel containing ethidium bromide and visualized under UV-light.

### Micro electrode array

Micro electrode array (MEA) is a noninvasive method for neuronal network cellular activity measurement in research designs with a long term monitoring (Pine, 1980). MEAs allow the observation of the network activity of neuronal cells, including hESC-derived neuronal cells (Heikkilä et al., 2009). They can be used for monitoring of the neuronal network activity and plasticity, action potential measurements, drug screening, and toxicological studies (Johnstone et al., 2010).

To analyse the neuronal activity, a single cell suspension was also replated in NDM on 0.05% polyethyleneimine and 20 µg/ml human laminin coated MEA plates (*n* = 8, 6wellMEA200/30iR-Ti, Multi Channel Systems, Germany) containing custom-made poly(dimethylsiloxane, PDMS) culturing chambers (Kreutzer et al., 2012). These chambers have capacity of 200 µl of medium/well and the cell attachment area is limited to 7 mm^2^.

As with the 48-well plates, three days after cell seeding, the medium in the 1/3 of MEA wells was replaced with artificial CSF, human CSF, or NMD, respectively. For the measurements, each MEA dish was placed in the MEA amplifier and let to stabilize for few minutes. The temperature was maintained at 37°C with a TC02 temperature controller (Multi Channel Systems). Each recording lasted 5 to 10 min. The measurements were controlled with MC_Rack software (Multi Channel Systems) and 20 or 50 kHz sampling rates were used. The first activity measurements were performed after one week's culture in MEA and thereafter twice a week for three weeks. MC_Rack and NeuroExplorer (Nex Technologies, Littleton, MA) softwares were used for the data analysis. For the spiking rate analysis, the spikes were sorted from each MEA well against the standard deviation of background times 5. Thereafter, the sorted spikes were calculated. The results were normalized against the baseline (three days on MEA) activity and plotted as a graph.

### Immunocytochemical staining

Immunocytochemical stainings were performed as previously described (Lappalainen et al., 2010; Sundberg et al., 2009). Each week the remaining cells (at least 5 wells/condition after collection of BrdU and RT-PCR samples) were fixed with cold 4% paraformaldehyde for 15 min. Next, a 45 min blocking was performed with 10% normal donkey serum (NDS), 0.1% Triton-X, and 1% bovine serum albumin (BSA) in phosphate buffered saline (PBS). Thereafter the cells were washed with 1% NDS, 0.1% Triton-X, and 1% BSA in PBS and incubated overnight at 4°C with primary antibodies diluted to the same solution. The primary antibodies used were rabbit anti-MAP-2 (1:800, Chemicon, Temecula, CA), mouse anti-β-tubulin_3_ (1:1200, Sigma–Aldrich), sheep anti-GFAP (1:800, R&D Systems), rabbit anti-BLBP (1:800, Chemicon), and mouse anti-GalC (1:400, Chemicon). Triton-X was omitted from the protocol when staining with anti-GalC. The following day the cells were washed 3 times with 1% BSA in PBS and incubated with AlexaFluor-488 or AlexaFluor-568 secondary antibodies conjugated to mouse, sheep, or rabbit antibodies (Invitrogen). Finally, the cells were washed 3 times with PBS, 2 times with phosphate buffer, and mounted with Vectashield including 4′6-diamidino-2-phenylindole (DAPI, Vector Laboratories, UK). The imaging was accomplished with Olympus microscope (IX51, Olympus, Finland) equipped with a fluorescence unit and a camera (DP30BW, Olympus). For manual cell counting, at least five pictures with magnification of 100 were taken from each well.

### Luminex

The levels of six growth factors including brain-derived neurotrophic factor (BDNF), nerve growth factor beta subunit (B-NGF), epidermal growth factor (EGF),bFGF, platelet-derived growth factor BB (PDGF-BB), and vascular endothelial growth factor A (VEGF-A) in the pooled CSF was analyzed with the Procarta® Immunoassays Kit for bodily fluid samples (Panomics, Affymetrix Inc., Santa Clara, USA) according to the manufacturer's protocol. Briefly, mixed antibody beads were added to each well as well as standards and supernatant and incubated for 60 minutes at RT. Detection antibody solution was added and the plate was incubated for 30 minutes. Thereafter, streptavidin-PE solution was added and incubated for 30 min. Finally, reading buffer was added and samples were run with Luminex instrument (Bio-Plex™ 200 System, based on Luminex® xMAP Technology). All the data were collected and analyzed using Bio-Plex manager software version 6.0 (Bio-Rad laboratories). Standards and CSF were run in duplicates. Five parameter regression models were used to calculate the molecules concentrations. The percent recovery of standards ranged from 90 to 110% and that was used detection limit for each protein. Detection limit for each molecule as follows: 19.5 pg/ml for BDNF, 3.3 pg/ml for B-NGF, 0.6 pg/ml for EGF, 5.2 pg/ml for FGF-2, 1.7 pg/ml for PDGF-BB, 1.2 pg/ml for VEGF-A. The samples had the percent of coefficient of variation (%CV) less than 7%.

### Statistics

Data from BrdU and time-lapse imaging analysis were statistically analyzed with nonparametric Kruskal–Wallis test and Mann–Whitney U-test with Bonferroni correction, using SPSS version 17.0 (SPSS Inc., IL, USA).

## Supplementary Material

Supplementary Material
