# Supplementary material for: Healthy human CSF promotes glial differentiation of hESC-derived neural cells while retaining spontaneous activity in existing neuronal networks
Source: Biol Open. 2013 May 13;2(6):605–12. doi: 10.1242/bio.20134648 (PMC3683163; doi:10.1242/bio.20134648)
Supplement: Supplementary Material [file supp_2_6_605__index.html]

Healthy human CSF promotes glial differentiation of hESC-derived neural cells while retaining spontaneous activity in existing neuronal networks — Healthy human CSF promotes glial differentiation of hESC-derived neural cells while retaining spontaneous activity in existing neuronal networks — Supplementary Material 

# Healthy human CSF promotes glial differentiation of hESC-derived neural cells while retaining spontaneous activity in existing neuronal networks

## bio.20134648 Supplementary Material

**Files in this Data Supplement:**

- Supplementary Material - Heikki Kiiski et al. doi: 10.1242/bio.20134648
