# Supplementary material for: Healthy human CSF promotes glial differentiation of hESC-derived neural cells while retaining spontaneous activity in existing neuronal networks
Source: Biol Open. 2013 May 13;2(6):605–12. doi: 10.1242/bio.20134648 (PMC3683163; doi:10.1242/bio.20134648)
Supplement: Supplementary Material [file supp_bio.20134648_bio.20134648-s1.pdf]

Biology Open

Supplementary Material

Heikki Kiiski et al. doi: 10.1242/bio.20134648

Table S1. The primers used for RT-PCR analysis.

Gene	Primer sequence	T_a(℃)	Size (bp)
Oct-4	*F* 5′-CGTGAAGCTGGAGAAGGAGAAGCTG-3′	55	245
	R 5′-AAGGGCCGCAGCTTACACATGTTC-3′	55	
α-fetoprotein	*F* 5′-GCTGGATTGTCTGCAGGATGGGGAA-3′	60	216
	R 5′-TCCCCTGAAGAAAATTGGTTAAAAT-3′	60	
Brachyury/T	*F* 5′-GCTTCAAGGAGCTCACCAAT-3′	63	425
	R 5′-CACCGCTATGAACTGGGTCT-3′	63	
Musashi	*F* 5′-AGCTTCCCTCTCCCTCATTC-3′	59	161
	R 5′-GAGACACCGGAGGATGGTAA-3′	60	
Nestin	F 5′-CAGCTGGCGCACCTCAAGATG-3′	68	208
	R 5′-AGGGAAGTTGGGCTCAGGACTGG-3′	74	
Pax-6	*F* 5′-AACAGACACAGCCCTCACAAACA-3′	60	174
	R 5′-CGGGAACTTGAACTGGAACTGAC-3′	60	
MAP-2	*F* 5′-AATAGACCTAAGCCATGTGACATCC-3′	60	132
	R 5′-AGAACCAACTTTAGCTTGGGCC-3′	60	
BLBP	*F* 5′-CGCTCCTGTCTCTAAAGAGGGG-3′	60	594
	R 5′-TGGGCAAGTTGCTTGGAGTAA-3′	60	
GFAP	*F* 5′-GCTCGATCAACTCACCGCCAACA-3′	60	207
	R 5′-GGGCAGCAGCGTCTGTCAGGTC-3′	60	
NG2	*F* 5′-CTCCTGCTCTACCGTGTGGTG-3′	60	168
	R 5′-GGGCATCTCATGCTCATACAG-3′	60	
Nkx6.2	*F* 5′-GAGAGCCAGGTGAAGGTCTG-3′	60	221
	R 5′-TTCGAGGGTTTGTGCTTCTT-3′	60	
PDGFR	*F* 5′-CTATCCACACTGTCAAACAGGTTG-3′	60	452
	R 5′-TCTGCTGGACTGAGAAGTTTCATC-3′	60	
Sox10	*F* 5′-ATCCAGGCCCACTACAAGAG-3′	60	238
	R 5′-GAAGTCGATGTGAGGCTTCC-3′	60	
GAPDH	*F* 5′-AGCCACATCGCTCAGACACC-3′	55	302
	R 5′-GTACTCAGCGGCCAGCATCG-3′	55	
